# Dickkopf-1 and Amphiregulin as Novel Biomarkers and Potential Therapeutic Targets in Hepatocellular Carcinoma

**Published:** 2019-07-01

**Authors:** Abeer E. Awad, Mohamed A. Ebrahim, Laila A. Eissa, Mamdouh M. El-Shishtawy

**Affiliations:** 1 Department of Biochemistry, Faculty of Pharmacy, Mansoura University, Mansoura, 35516, Egypt; 2 Oncology Centre, Faculty of Medicine, Mansoura University, Mansoura, 35516, Egypt

**Keywords:** HCC, Dickkopf-1, Amphiregulin

## Abstract

**Background: **Hepatocellular carcinoma (HCC) is a highly fatal tumor which represents a major health problem worldwide. Due to asymptomatic nature of HCC, most patients present with the progressive stage of disease, so, unfortunately, there are no effective therapies. Existing techniques for HCC surveillance and diagnosis lack the required accuracy. Therefore, searching for new diagnostic and/or therapeutic tools could improve patient survival. This study aimed to estimate the diagnostic role of Dickkopf-1 (DKK1) and amphiregulin (AREG) and to find out their correlation with different clinicopathological parameters in HCC patients.

**Materials and Methods:** Serum levels of DKK1 and AREG in 55 HCC patients, 20 cirrhotic patients, and 15 healthy subjects as control group were measured using the ELISA technique.

**Results:** Both of DKK1 and AREG showed a significant increase in the HCC group compared to cirrhotic and healthy groups. DKK1 at a cutoff point of 8.92 ng/ml showed that the area under the curve (AUC) was 0.826 with 87.3% sensitivity and 82.9% specificity. DKK1 showed a significant correlation with tumor size, liver dysfunction, and poor performance status in HCC patients. AREG at a cutoff point of 8.74 pg/ml showed a sensitivity of 74.5% but low specificity (47.1%). AREG showed a significant correlation with portal vein thrombosis and tumor metastasis in HCC patients.

**Conclusion:** Serum DKK1 could be a diagnostic biomarker for HCC. Both of DKK1 and AREG may play significant roles in tumor progression and may offer promising therapeutic targets in HCC patients.

## Introduction

 HCC is a prevalent worldwide cancer and has a high fatality rate which makes it the second most mortal malignancy^1^. This miserable outcome is due to absence of accurate markers for early diagnosis which makes the available therapeutic tools of limited survival benefit^   2 ^. 

The current available tools for HCC screening are liver ultrasound in combination with serum alpha-fetoprotein (AFP). Liver ultrasound depends to a great extent on operator’s experience. In addition, the cirrhotic background may impede the early identification of small tumors by ultrasound^   3 ^. AFP can be falsely raised in chronic liver diseases^   4 ^. Another limitation of AFP is its low sensitivity as over 45% of HCC cases may have normal AFP levels^5^. Therefore, current guidelines for American Association for Study of Liver Diseases recommend ultrasound as a basic modality for HCC screening with or without AFP^   6 ^ .

Precise knowledge about different molecular pathways involved in initiation or progression of HCC could reveal more effective diagnostic and/or treatment tools.

Wnt and epidermal growth factor receptor (EGFR) pathways are two prominent pathways which are involved in multiple cellular functions and their aberrant regulation is a prevalent theme in cancer biology^7^^,^^8^.

DKK1 acts as inhibitory ligand of Wnt/β-catenin pathway. This signaling pathway is activated upon binding of Wnt protein to its specific receptor Frizzled (FZ) and its co-receptor low density lipoprotein receptor related protein 5 or 6 (LRP5/6), which leads to cytoplasmic β-catenin accumulation and activation of target genes^   9 ^. DKK1 binds to LRP5/6 and prevents the formation of Frizzled (FZD)-Wnt-LRP5/6 complex^   10 ^. DKK1 could also inhibit this pathway by promoting LRP5/6 internalization upon interaction with kremen proteins^   11 ^.

Furthermore, DKK1 is a downstream target of the Wnt/β-catenin pathway performing a regulatory effect on this pathway through negative feedback loop^   12 ^. In accordance with this suppressive function, it has been found to be silenced by hypermethylation in some cancer types; however, it is upregulatd in others as multiple myeloma and HCC. This may refer to disrupted feedback loop or that the suppressive effect of DKK-1 is limited and functions only until certain point of saturation^   13 ^. Another model suggested that DKK1 can stimulate proliferation in cancer cells through AKT signaling independent of wnt pathway ^   14 ^^.^

Amphiregulin (AREG) is a ligand of the EGFR which has an essential role in cell proliferation, survival, and migration.

 AREG is synthesized as a membrane anchored precursor protein (Pro-AREG) which undergoes proteolytic processing by tumor necrosis factor-alpha converting enzyme (TACE). Soluble AREG is then secreted to interact with EGFR triggering various signaling pathways such as PI3K/AKT, Ras/MAPK ^15^^, ^^16^ . AREG expression in normal liver is very low^   17 ^, but it is greatly increased upon liver injury providing an important pro-regenerative function ^18^^,^^19^ . However, an exacerbated reparative response may have deleterious consequences as AREG induces an autocrine loop sustaining the survival features of HCC cells ^   20 ^.

 Our study aims to evaluate the diagnostic performance of DKK1 and AREG as serum biomarkers in HCC and finding out their correlation with the clinicopathological parameters of HCC patients.

## MATERIALS AND METHODS

 Serum levels of AFP, DKK1 and AREG were evaluated in HCC and cirrhotic patients in addition to healthy controls. From May 2014 to July 2015, 55 HCC patients (46 males and 9 females; with a mean age±SD = 56.61±8.26) from Oncology Center, Mansoura University, Mansoura, Egypt were enrolled in the study. A full medical history and accurate clinical examination were performed for all HCC patients. HCC cases were defined on the basis of abdominal ultrasound and serum AFP, and then confirmed by computed tomography scan or magnetic resonance imaging of the abdomen and biopsy when needed. HCC patients are classified according to Barcelona Clinic Liver Cancer (BCLC) staging system (Table 1). We also recruited a group of 20 cirrhotic patients (14 males and 6 females; with a mean age±SD = 55.75±7.70) from inpatients clinic of Mansoura university Hospital, Mansoura University, Mansoura, Egypt. Child-Pugh classification is used to estimate the degree of liver cirrhosis in all patients (Table 1). Patients with a history of other types of solid tumors, mixed HCC-cholangiocarcinoma as well as advanced medical comorbidity were precluded from the study. HCC patients receiving previous treatment with chemotherapy were also excluded to avoid its effect on the markers of the study. A control group of 15 apparently healthy subjects (12 males and 3 females, mean age±SD = 51.80±11.81) with normal liver biochemistry and no evidence of viral hepatitis was also involved. All groups were statistically matched in terms of age and sex (Table 1). An informed consent was obtained from all subjects in the study, and the study was approved by Faculty of Pharmacy, the Ethics Committee of Mansoura University.

**Blood samples collection and handling**

Peripheral blood samples from all subjects in the study were collected into two sections. The first monovette containing anticoagulant for blood picture investigation. The other monovette with no additives were left to clot for 20 min, and then centrifuged at 3000 rpm for 10 min. The produced serum was divided into two portions. The first portion is used for liver function test (ALT, AST activities, albumin and bilirubin levels), while the other is frozen and stored at −80°C until used.

**Measurements of study parameters**

Serum AFP was measured using a commercially available DS-EIA-AFP ELISA kit from (DSI S.r.l. Saronno, Italy). Serum DKK1 and AREG were measured using commercially available kits from (MyBiosource, San Diego, CA, USA) according to the manufacturer`s recommendations.

**Statistical analysis**

Data were statistically analyzed with SPSS version 21. The normality of data was first tested with one-sample Kolmogorov-Smirnov test. Chi-square test was used to test the association between categorical variables which were presented as number and percent. Continuous variables were presented as mean ± SD (standard deviation) for parametric data and Median for non-parametric data. In case of comparing between the two groups, we used Student *t-*test for parametric data and Mann–Whitney test for non-parametric data. In case of comparing the means of more than two groups, ANOVA test was used for parametric data, while Kruskal Wallis test was used for comparing the median of more than two groups for non-parametric data. Sensitivity and specificity at different cutoff points were tested by receiver operating characteristic (ROC) Curve. A value of P˂0.05 was considered significant. 

## Results

 Characteristics of cirrhotic and HCC patients are illustrated in Table (1). 

**Table 1:** Characteristics of hepatocellular carcinoma (HCC) and cirrhotic patients

**Items**	**HCC (n=55)**		**Cirrhosis (n=20)**	
Age (mean ± SD)	56.61 ± 8.26		55.75 ± 7.70	
Items	n	%	n	%
Sex				
Male	46	83.6	14	70.00
Female	9	16.4	6	30.00
Ascities				
Absent	31	56.4	2	10.0
Mild	15	27.3	5	25.0
Moderate	7	12.7	4	20.0
Marked	2	3.6	9	45.0
Child-Pugh classification				
A	35	63.6	9	45.0
B	13	23.6	6	30.0
C	7	12.7	5	25.0
Virology				
HCV negative	10	18.2	6	30.0
HCV positive	45	81.8	14	70.0
Performance status				
1	29	52.7		
2	19	34.5		
3	7	12.7		
Portal vein thrombosis				
Patent	39	70.9		
Thrombosed	16	29.1		
Metastasis				
Absent	24	43.6		
Present	31	56.4		
BCLC				
A	4	7.3		
B	16	29.1		
C	28	50.9		
D	7	12.7		
Number of lesions				
single	13	23.6		
bifocal	5	9.1		
multifocal	37	67.3		

n: Number of patients, HCV: Hepatitis C virus, BCLC: Barcelona clinic liver cancer staging

Comparison between laboratory data of HCC, cirrhotic and control groups are shown in Table (2).

**Table 2:** Laboratory data of HCC and cirrhotic patients as compared to control group

**Parameter**	**Control (n=15)**	**Cirrhosis (n=20)**	**HCC (n=55)**
AST activity (U/L)	24 (15 - 30)	67.5 (17 – 259)	89.7 (27.6 – 364.69) $
ALT activity (U/L)	24 (12 - 33)	33.5 (17 - 168)	62.53 (13.35 - 206.5) $
Total bilirubin concentration(mg/dl)	0.8 (0.5 – 1)	2.30 (0.7 - 30)	1.87 (0.71 – 17) $
Albumin concentration (g/dl)	4.60±0.46	2.45±0.67	3.42±0.73 $*
Hb concentration (g/dl)	14.17±1.64	10.715±1.91	12.37±2.07$*
WBCs count (x 10^3^/μl	6.00 (5.40 – 10.20)	7.20 (2.50 - 37.59)	6.63 (2.01 – 18.49)
Platelet count (x 10^3^/μl)	311 (199.9 – 402.3)	101 (26 – 231)	106.10 (11 – 402) $
AFP (ng/ml) Median (range)	7.63 (6.03 – 9.24)	45.39 (8.23 – 206.30) $	206.38 (5.93 – 478.08) $
DKK1 (ng/ml) Mean ± SD	5.88 ± 2.53	8.57 ± 3.06 $	11.22 ± 2.55 $*
AREG (pg/ml) Median (range)	9.33 (3.43 – 24.19)	9.17 (3.43 – 19.59)	14.27(5.46 – 43.44) $*

Parametric data are represented as mean ± SD while non-parametric data are represented as median (range). AST: Aspartate aminotransferase, ALT: Alanine aminotransferase, Hb: Hemoglobin, WBCs: White blood cells, AFP: Alpha fetoprotein, DKK1: Dickkopf-1, AREG: Amphiregulin, SD: Standard deviation, n = Number of subjects in each group,

$ = Significant against control group at p<0.05,

* = Significant against cirrhotic group at p<0.05.

**Table 3:** Receiver-operating characteristic (ROC) curves for detection of cutoff values of HCC prediction

**Item**	**AUC**	**95% Confidence Interval**		**Cut off point**	**Sensitivity**	**Specificity**	**PPV**	**NPV**
		**Lower limit**	**Upper limit**					
DKK1 (ng/ml)	0.826	0.729	0.923	8.92	87.3%	82.9%	88.89	80.56
AREG (pg/ml)	0.695	0.569	0.855	8.74	74.5%	47.1%	68.3	53.3

HCC: Hepatocellular carcinoma, DKK1: Dickkopf-1, AUC: Area under the curve, AREG: Amphiregulin, PPV: Positive predictive value, NPV: Negative predictive value

HCC patients showed a significant increase in serum DKK1 and AREG levels as compared to cirrhotic patients and control groups (P<0.05). Serum DKK1 was significantly higher in cirrhotic patients as compared to control groups. On the other hand, cirrhotic patients showed a non-significant increase in serum AREG level in comparison with control groups. 

**Figure 1:**
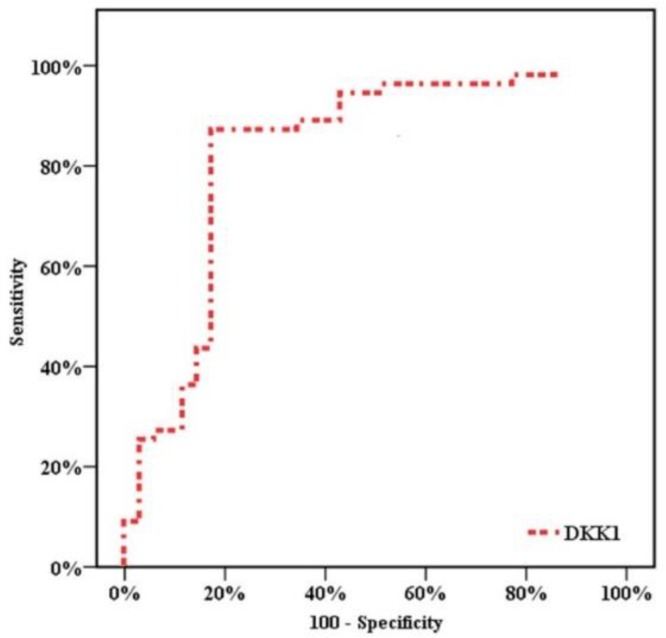
Receiver Operating Characteristic (ROC) curve for Dickkopf-1 (DKK1)

ROC curves showed that the optimum diagnostic cut off point for DKK1 was 8.92 ng/ml (AUC 0.826, sensitivity 87.3%, speciﬁcity 82.9%, positive predictive value (PPV) = 88.89, negative predictive value (NPV) = 80.56). (Table 3, Figure 1)

**Figure 2:**
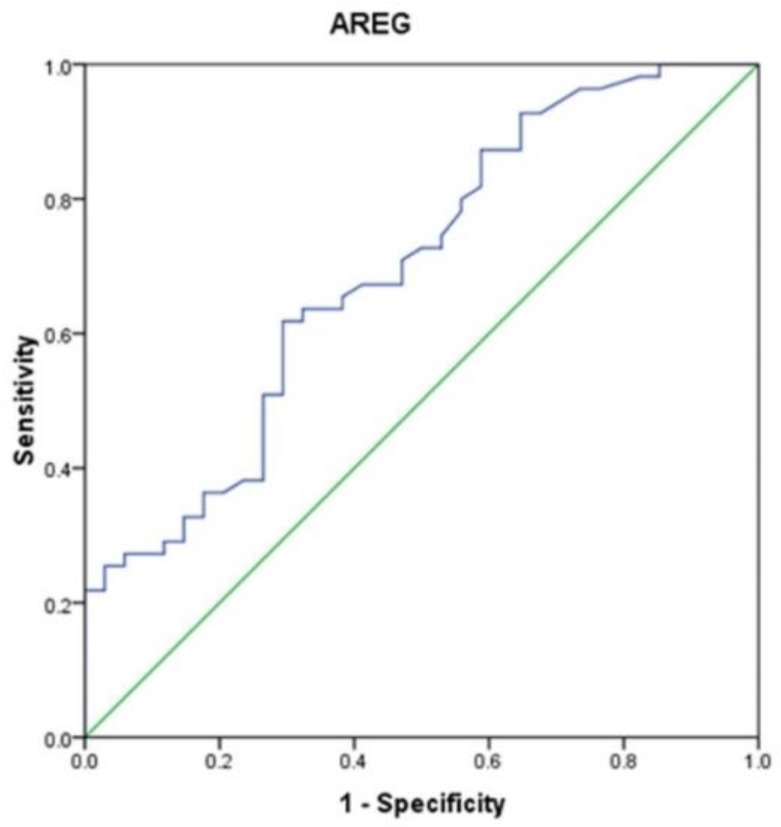
Receiver operating characteristic (ROC) curve for amphiregulin (AREG)

While AREG at a cut-off point of 8.74 pg/ml, showed an AUC = 0.695, sensitivity of 74.5% but with low specificity 47.1% (Table 3, Figure 2).

Correlation between serum DKK1 and liver functions in HCC patients revealed a significant positive correlation between serum DKK-1 and serum AST. In addition, significant negative correlation was found between serum DKK1 and serum albumin in HCC patients. Correlation between serum AREG and liver functions in cirrhotic and HCC patients did not show any significant correlation (Table 4).

Correlation between serum AREG and blood picture parameters in HCC patients showed a significant negative correlation with white blood cells count (Table 4).

**Table 4:** Correlation between study markers and patients parameters in cirrhotic and HCC groups

**Parameters**	**Cirrhotic patients**			**HCC patients**
	**R**	**p**	**R**	**P**
DKK1				
ALT	0.218	0.355	0.170	0.225
AST	0.181	0.445	0.305	0.028
Total bilirubin	0.432	0.065	-0.017	0.905
Albumin	-0.281	0.244	-0.346	0.014
Hb concentration (g/dl)	0.045	0.849	-0.176	0.209
WBCs count (x 10^3^/μl)	0.487	0.034	-0.004	0.976
Platelets count (x 10^3^/μl)	0.257	0.273	-0.086	0.539
AREG				
ALT	0.226	0.353	0.058	0.675
AST	0.198	0.417	0.114	0.416
Total bilirubin	0.165	0.513	-0.104	0.456
Albumin	-0.231	0.357	-0.076	0.596
Hb concentration (g/dl)	0.003	0.991	-0.140	0.313
WBCs count (x 10^3^/μl)	0.200	0.411	-0.273	0.046
Platelets count (x 10^3^/μl)	-0.137	0.577	-0.145	0.295

r= Correlation coefficient, HCC: Hepatocellular carcinoma, DKK1: Dickkopf-1, AREG: Amphiregulin, ALT: Alanine aminotransferase, AST: Aspartate aminotransferase, Hb: Hemoglobin, WBCs: white blood cells

**Figure 3:**
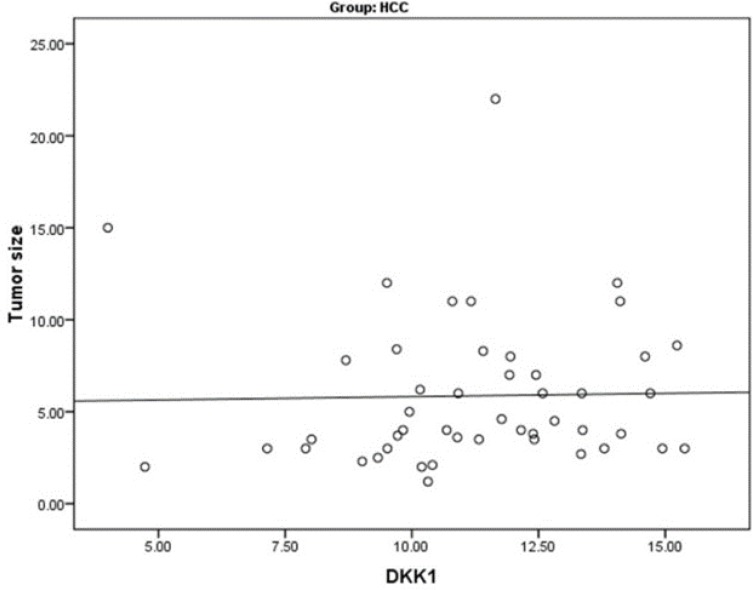
Significant positive correlation between serum Dickkopf-1(DKK1) level and tumor size in hepatocellular carcinoma

Furthermore, correlation between tumor characteristics of HCC patients and studied markers showed significantly positive correlation between serum DKK1 and tumor size (Figure 3). Serum DKK1 was significantly related to the performance status of HCC patients. On the other hand, serum AREG showed a significant relation to portal vein thrombosis and metastasis (Table 5).

**Table 5:** Relation of serum DKK1 and AREG concentrations to some tumor characteristics in HCC group

		**Serum DKK1 concentration**			**Serum AREG concentration**	
		**Mean** ** ±** ** SD**	**P**	**Median**	**Range**	**P **
Performance status	1	9.81 ±2.2	<0.001	14.44	5.96 - 36.68	0.305
	2	12.13 ±1.9		13.73	6.98 – 43.44	
	3	14.59±0.8		8.33	5.46 – 38.88	
Ascites	Absent	11.3 ± 2.2	0.775	14.44	5.96 – 43.44	0.557
	Mild	10.7 ±2.9		13.73	6.98 – 26.39	
	Moderate	11.4 ±2.8		8.33	5.46 - 26.81	
	Marked	12.4 ±4.0		26.07	13.26 - 38.88	
Metastasis	Absent	11.7± 2.2	0.442	17.18	6.64 - 43.44	0.024
	Present	11.0± 2.6		10.26	5.46 - 31.88	
Portal vein thrombosis	Absent	11.2 ± 2.4	0.788	15.42	5.46 – 43.44	0.012
	Present	11.0 ± 2.9		12.59	7.65 – 25.96	
BCLC	A	10.9 ±1.6	0.966	14.53	10.18 – 18.45	0.489
	B	11.0 ± 2.7		14.02	5.96 – 36.68	
	C	11.2 ± 2.5		14.68	6.98 – 43.44	
	D	11.5 ±3.1		8.33	5.46 – 38.88	

DKK1: Dickkopf-1, AREG: amphiregulin, HCC: hepatocellular carcinoma, BCLC: Barcelona clinic liver cancer staging

In addition, DKK1 levels in HCV-positive HCC patients (mean±SD=11.4±2.5) were significantly higher than those without HCV (mean±SD=7.9±3.1) (p=0.031). However, no relation was found between serum AREG and virology status in HCC patients.

Our results showed no relation between Child-Pugh classification and neither serum DKK1 nor AREG levels in patients with liver cirrhosis or HCC.

## Discussion

 HCC is a major cause of cancer-related death due to lack of early detection methods, ineffective therapies and frequent recurrence or metastasis^21^. Late diagnosis leads to only a small percentage of patients to be suitable for effective therapeutic options as liver transplantation, resection or local ablation therapy^   22 ^. 

Our study depended on assessment of the diagnostic performance of serum DKK1 and AREG in HCC detection in addition to their correlation with different clinicopathological parameters in HCC and cirrhotic patients.

DKK1 showed AUC of 0.826 with sensitivity 87.3%, specificity 82.9%, PPV 88.89 and NPV 80.56. Therefore, serum DKK1 could be used as a diagnostic biomarker for HCC.

Serum DKK1 level in our cases was significantly higher in HCC patients than cirrhotic and control groups. This result was in accordance with many reports ^23^^-^^25^ . In addition, there was a significant difference in serum DKK1 level between cirrhotic and control groups.

Shen, Fan^24^ and Erdal, Gül Utku^26^ demonstrated that serum DKK1 was statistically similar between cirrhotic and control groups. Tung, Mak ^27^ and Kim, Park^23^ also showed statistically similar serum DKK1 level in spite of significantly higher DKK1 gene expression in cirrhotic patients. In agreement with our study, Mohamed, Barakat^28^ showed a significantly higher serum DKK1 level in cirrhotic patients than healthy group. In addition, Kim, Park^23^ found a significant correlation between serum DKK1 level and necroinflammatory activity. This may suggest the early secretion of DKK1 in serum in case of cirrhosis as a preneoplastic condition.

Our results showed a significant positive correlation between serum DKK1 and tumor size. The effect of DKK1 on cell proliferation and tumor size remains controversial. Kim, Park^23^ found no correlation between serum DKK1 level and tumor size. However, Tung, Mak^27^ and Shen, Fan^24^ showed that serum DKK1 was significantly correlated with tumor size. DKK1 could increase cancer cell proliferation through binding to Cytoskeleton-associated protein 4 (CKAP4) independent of Wnt pathway^   29 ^.On the other hand, Glaw, Skalak^30^ found significantly increased vascular density and draining vessel diameter in DKK1-treated rats. A recent study showed that DKK1 increased angiogenesis through upegulation of vascular endothelial growth factor receptor 2 in a wnt-independent manner^   31 ^. Moreover, DKK1 promoted the growth of tumor cells in animal models via enhanced vasculogenic mimicry formation^   32 ^. Vasculogenic mimicry is a characteristic feature of highly aggressive tumors which means de novo generation of microvascular channels independent of endothelial cells. Therefore, DKK1 may enhance tumor growth via direct effect on cellular proliferation and/or indirectly through promoting tumoral angiogenesis or vasculogenic mimicry in the tumor microenvironment. 

We found no correlation between DKK1 level and BCLC stages. This result was in line with that of Shen, Fan^24^. On the other hand, Kim, Park^23^ reported significantly elevated DKK1 level in BCLC stage C-D than BCLC stage A-B.

We found that DKK1 levels in HCV-positive HCC patients were significantly higher than those without HCV. Gene expression profile revealed that DKK1 gene is highly expressed in HCV-related HCC^33^. 

Significant positive correlation was found between serum DKK1 and AST but not ALT in HCC patients. Furthermore, a significant negative correlation between serum DKK1 and serum albumin in HCC patients was illustrated as well. In the majority of mild to moderate chronic hepatitis C, ALT activity was increased compared to AST, but as fibrosis advances, AST activity was increased, and then AST/ALT ratio consequently increased. Several studies have shown that the AST/ALT ratio is typically < 1 in patients with chronic hepatitis, but with progression to cirrhosis, the ratio often increases to over 1  ^34^^,^^35^ . This may be due to the release of mitochondrial fraction of AST by progressive damage ^   36 ^ or decreased AST clearance by sinusoidal cells^   37 ^. We then suggest that DKK-1 may have certain relation to liver damage and deterioration of its synthetic ability in HCC patients and that targeting DKK1 may have positive effects on liver functions during the hepatocarcinogenesis process.

AREG expression in normal liver is very low; however, its level is markedly increased upon liver injury, providing a prominent regenerative role in liver tissues  ^17^^,^^19^ . However, it was found that AREG stimulates connective tissue growth factor expression^   38 ^ and extracellular matrix-producing cells proliferation^   39 ^. This shows that excessively active reparative response by AREG participates in liver fibrosis.

There is a lot of evidence that refers to the involvement of AREG in hepatocarcinogenesis. HCC cells were found to overexpress and secrete AREG producing an autocrine stimulation loop to achieve self-sufficiency in growth signals^   20 ^. AREG showed antiapoptotic effects through AKT and STAT-3 survival pathways, and was recently found to be involved in the activation of SOS-1 pathway in hepatoma cells ^   46 ^. 

Serum AREG level in our HCC cases was significantly higher than that of cirrhotic patients and healthy group. Serum AREG level in cirrhotic patients was not significantly increased as compared to the healthy group. This was in agreement with that of Han, Bai ^47^ who showed that serum AREG levels were upregulated in HCC patients and could be used as a candidate biomarker for HCC diagnosis.

In our results, the AUC of AREG was 0.695. At a cut-off point of 8.74 pg/ml, AREG showed a sensitivity of 74.5%, but with low specificity 47.1%.

Our study revealed a significant relation between serum AREG level and portal vein thrombosis as well as metastasis in HCC patients. AREG as a downstream target of yes associated protein (YAP) found to be a key mediator of YAP responses, involving cell proliferation and migration^   48 ^. Castillo, Erroba^20^ found that AREG promotes anchorage-independent growth of HCC cells which is a characteristic feature of highly aggressive and metastatic phenotype of cancer cells. Moreover, many studies reported AREG role in promoting the ability of tumor cells to migrate in different cancer types ^49^^-^^51^ .

Interestingly, although AREG level was significantly higher in HCC patients than cirrhotic and control groups, the presence of portal vein thrombosis or metastasis in HCC patients showed significantly lower serum AREG level as compared to non-metastatic patients and those without portal vein thrombosis. Higginbotham, Beckler^52^ identified a new signaling pattern for EGFR ligands through what is called exosomes. These extracellular microvesicles can mediate cell-cell communication through transfer of proteins and RNAs from origin to recipient cell^   53 ^. Tumor-associated exosomes play a key role in tumor progression as they cause amplification of oncogenic signals and mediate distant signaling which leads to tumor spread ^   54 ^.

An elegant study made by Higginbotham, Beckler^52^ reported about 24 membrane-stable AREG molecules per a single exosome. Moreover, this study revealed a five-fold increase in the invasive ability of cancer cells receiving AREG exosomes over those receiving an equivalent amount of recombinant AREG. They postulated that relative membrane stability in addition to compact packaging make exosomes act as a multivalent EGFR ligand which facilitates the aggregation of EGFR in the recipient cells. In addition, tumor-derived exosomes may act locally on surrounding microenvironment^   55 ^ or secreted into blood stream and settle in distant organs providing an attractive environment for circulating EGFR-overexpressing tumor cells to reside (forming a metastatic niche)^56^. 

Furthermore, another recent study showed the role of AREG exosomes in bone metastasis induction in non-small cell lung cancer patients and that targeting these exosomes may improve the therapeutic strategy^   57 ^^.^ According to these findings, metastatic HCC patients may show lower free AREG in serum as the translocation of AREG within exosomes may be a predominant form during metastasis due to higher invasive capacity. However, this point needs further investigation.

On the other hand, Tanaka, Nishioka ^58^ showed that ectodomain shedding process yields two types of signaling pathways. The extracellular signaling elicited by the autocrine, juxtacrine and paracrine interactions of soluble AREG with EGFR and the intracellular signaling which increased cell migration via the translocation of unshed pro-AREG to nuclear envelope. They revealed that disrupted coordination between these two pathways may lead to cancer metastasis. Therefore, shifting toward the intracellular signaling may direct the cancer cell in the way of migration and invasion. 

 Accordingly, this unexpected inverse relation between serum AREG level and metastasis as well as vascular invasion raise the question of whether there are other forms than soluble AREG that could be implicated in dissemination of tumors from liver to distant organs. We also suggest that decreased AREG levels in serum of metastatic HCC patients may be accompanied by an increase in other AREG forms in tissues. Investigation of different AREG forms in the future studies may reveal if tissue targeting is a possible tool to prevent disease progression. 

## CONCLUSION

 Altogether, we propose that beside DKK1 role in HCC diagnosis, it could be a promising therapeutic target in HCC patients. Serum AREG relation to metastasis suggests that tissue targeting of different AREG forms may represent a novel trend to prevent tumor progression and metastasis in HCC patients. 
